# Soil function, properties and plant diversity–biomass patterns differ between grazed and non-grazed steppe ecosystems

**DOI:** 10.3389/fpls.2025.1597590

**Published:** 2025-07-08

**Authors:** Jia Mi, Qianju Wang, Jun Ou, Jing Shi, Haiyan Pang, Ziheng Feng, Yongfei Bai

**Affiliations:** ^1^ Shanxi Key Laboratory for Ecological Restoration of Loess Plateau, Institute of Loess Plateau, Shanxi University, Taiyuan, China; ^2^ Field Scientific Observation and Research Station of the Ministry of Education for Subalpine Grassland Ecosystem in Shanxi, Ningwu, China; ^3^ College of Environment and Resources Sciences, Shanxi University, Taiyuan, China; ^4^ State Key Laboratory of Vegetation and Environmental Change, Institute of Botany, Chinese Academy of Sciences, Beijing, China; ^5^ College of Resources and Environment, University of Chinese Academy of Sciences, Beijing, China

**Keywords:** grazing and enclosure, plant-soil system, soil pH, biomass allocation, plant biodiversity

## Abstract

**Introduction:**

Grazing and enclosure are two major grassland management techniques, which are used to preserve plant variety, productivity, and ecosystem function.

**Methods:**

In order to compare plant diversity and ecosystem function under grazing and enclosure conditions, this study observed three typical grassland locations in southeast of Inner Mongolia via medium-scale line transect surveys.

**Results:**

Our results showed that soil nutrients in enclosed grasslands control the diversity of plant species and aboveground biomass (AGB), which in turn regulates the amount of belowground biomass (BGB) by allocation. Enclosure consistently enhanced AGB and plant height compared to grazing, while increasing the relative contribution of perennial grasses and forbs to productivity through functional group reorganization. However, biodiversity responses were site-specific, enclosure increased plant diversity at two sites but reduced it at another, revealing landscape-dependent results.

**Discussion:**

The grazing reshaped ecosystem regulation through three key changes: (1) the relationship between soil nutrients and AGB was inverted, and demonstrated a negative correlation between diversity and AGB, (2) established the trade-off of the effects of BGB on AGB driven by soil properties (soil nutrients and pH), (3) microbial community restructuring from dual nutrient-pH regulation to pH-dominated control and (4) grazing strengthened plant biomass-diversity linkages, while enclosure prioritized soil nutrient-plant diversity correlations. Crucially, grazing reversed the functional role of soil pH, from positive microbial community regulation in the enclosure area to negative effects, through soil microenvironment alteration. These results provided a framework in which management practices reorganize ecological networks. Enclosure strengthened soil nutrient-mediated plant-soil feedbacks, while grazing promoted pH-driven microbial selection and change of biomass allocation strategy. Meanwhile, the spatial variability of enclosure effects highlighted the importance of local environmental backgrounds for consequences of grassland management.

## 1 Introduction

Grassland ecosystems cover approximately 37% of the global land surface (O’Mara, 2012), making them the most extensive human-nature coupled system on Earth. They contribute about 40% of the global agricultural gross domestic product (GDP) (Herrero et al., 2013). Grasslands are vital for socio-economic development, providing key ecosystem services such as pasture supply, soil and water conservation, and wind and sand fixation (Li et al., 2021a). However, grasslands are also among the most fragile ecosystems, facing degradation in plant diversity, soil health, and ecosystem services due to human activities and extreme climate events (Filazzola et al., 2020). As part of the Eurasian steppe, the Inner Mongolian steppe serves as a critical ecological barrier in northern China. With its long history of grazing, it plays an indispensable role in supporting the local livestock economy (Yang et al., 2017). In recent decades, this region has experienced severe grassland degradation caused by overgrazing and poor management, resulting in reduced biodiversity and a decline in ecosystem functions (Fan et al., 2019). Therefore, understanding the resilience of grassland ecosystems to anthropogenic disturbances and promoting the restoration of biodiversity is essential for ensuring long-term sustainability.

The complex interrelationships among diverse ecological factors within the grassland ecosystem are intricately intertwined with its fundamental functions (Fan et al., 2019). These ecosystem functions encompass those facilitated by alterations in above-ground and underground ecological processes, including the accumulation of plant productivity, nutrient cycling, and the preservation of biodiversity (Wang et al., 2025). Grazing is an important type of utilizing grassland, primarily impacting the structure and function of the grassland ecosystem through the activities of herbivores (Zhang et al., 2023b). The impact of grazing livestock on grassland ecosystems is multifaceted, encompassing the processes of feeding, trampling, and excretion, and these activities can exert a significant influence on the productivity of grasslands and the composition and stability of their community structure (Wang et al., 2019). Furthermore, it will result in the modification of biomass allocation of the plant community (Zhou et al., 2020). Meanwhile, the physical and chemical properties of the soil, as well as the soil microbial community, are subject to alteration through the biological action of plant roots (Philippot et al., 2024). These changes will collectively modify the grassland ecosystem in a coupled manner (Liu et al., 2025). Nevertheless, the precise mechanisms by which grazing influences the structure and function of grassland ecosystems remain to be fully elucidated.

Grazing and enclosure are two key strategies in grassland management, both of which play a vital role in sustainable development and biodiversity conservation (Yang et al., 2022). According to the moderate disturbance hypothesis, reduced competitive exclusion and increased compensatory growth of plant communities under moderate disturbance, compared to ungrazed areas, supports higher community productivity and species diversity (Ramula et al., 2019). In contrast, overgrazing depletes species richness and ecosystem functionality by overharvesting and restricting nutrient availability (Herrero-Jáuregui and Oesterheld, 2018). Enclosure is widely regarded as one of the most cost-effective methods for restoring degraded grasslands, significantly improving vegetation characteristics and soil properties following grazing (Feyisa et al., 2017). Overgrazing can compact the soil and increase water evaporation due to livestock trampling, which negatively affects plant growth. Conversely, enclosure enhances the ability of plants to retain soil and water, promoting greater species richness and diversity (Fenetahun et al., 2021). However, in some arid regions, enclosure and grazing restrictions may have limited effects on plant diversity (Meissner and Facelli, 1999). Furthermore, soil biota, as a direct mediator of carbon, nitrogen, and phosphorus cycling, is crucial in driving plant diversity and ecosystem productivity (Liu et al., 2024). Grazing affects soil biota through multiple pathways, including alterations in plant community composition, soil physical properties, and nutrient dynamics (Martins et al., 2020).

As major approaches to grassland management, the mechanistic shifts in grazed grassland ecosystems are still not very clear. Compared with the enclosed grassland, what adjustments have occurred in the relationships and action pathways among various ecological factors in the ecosystem after grazing? In order to gain a deeper understanding of the effects of grazing and enclosure on these dynamics, the typical grasslands of Inner Mongolia were the subject of this study. We analyzed the characteristics of the plant community, the physicochemical characteristics of soil, and the features of the microbial community under both treatments by experimental comparisons of grazing and enclosure. Our study hypothesized that: (1) grazing reduces aboveground biomass, decreases the number of functional plant groups, lowers plant diversity, and alters the factors driving dominant plant diversity compared to enclosure; (2) enclosure promotes root growth and increases belowground biomass through microbial activity stimulation, while grazing enhances belowground biomass by altering resource allocation between plant organs, with the root system supporting aboveground growth; (3) soil pH is the primary factor influencing microbial community structure under grazing, while enclosure leads to microbial communities being influenced by both soil nutrients and pH due to reduced nutrient inputs.

## 2 Materials and methods

### 2.1 Overview of the study area

The experimental sites are situated in Hulunbuir City and Xilingol League, within the Inner Mongolia Autonomous Region, spanning a latitude range of 43.54° to 49.52°N, a longitude range of 116.56° to 120.03°E, and an elevation range of 640.3 to 1260 meters. The XL site, positioned in Xilinhot City with average annual precipitation of 290 mm, has a mid-temperate semi-arid continental monsoon climate. The EW site is located in the Ewenki Autonomous Banner of Hulunbuir City with average annual precipitation of 300 mm, while the CB site is in Chenbalhu Banner, Hulunbuir City with average annual precipitation of 320 mm, both regions characterized by a temperate continental climate. All three locations represent typical grassland ecosystems of the region.

The field survey results (**Table 1**) revealed that *Leymus chinensis* and *Stipa* spp. were the dominant species across the six sample sites, while approximately 95 additional species, such as *Carex duriuscula*, *Achnatherum sibiricum*, *Cleistogenes squarrosa*, *Filifolium sibiricum*, *Carex pediformis*, and *Adenophora stenanthina*, were also identified. These species represent 24 families and 61 genera, including Gramineae, Compositae, Leguminosae, and Rosaceae. The average vegetation coverage in the enclosure plots reached 77%, with an average height of 27.6 cm. In contrast, the grazing plots exhibited an average coverage of 46%, with a mean plant height of 10.0 cm.

**Table 1:** Plant community characteristics and dominant plant species of steppe in our experimental sites.

Sample sites	Longitude (°E)	Latitude (°N)	Number of species	Density (pcs/m^2)^	Coverage (%)	Mean height (cm)	Dominant species
Enclosed area XL	116.67	43.55	15	157.6	84.2	33.4	*Stipa capillata* *Achnatherum sibiricum* *Carex tristachya*
Grazed area XL	116.56	43.54	15	297.6	33.4	13.1	*Stipa capillata* *Leymus chinensis*
Enclosed area EW	119.67	48.49	48	246.8	74.2	21.8	*Stipa Baicalensis* *Leymus chinensis* *Filifolium sibiricum*
Grazed area EW	119.76	48.86	46	261.2	55.6	11.2	*Leymus chinensis* *Carex pediformis*
Enclosed area CB	120.02	49.52	29	642	72.6	27.7	*Leymus chinensis* *Carex duriuscula* *Adenophora stenanthina*
Grazed area CB	120.03	49.38	29	541.8	50	5.6	*Carex duriuscula* *Cleistogenes squarrosa* *Leymus chinensis*

### 2.2 Sample plot setting

The experiment was conducted in July, 2022 in a representative grassland area of the Inner Mongolia Autonomous Region. Three representative grassland locations were chosen for the study. At each location, two sample points were subjected to cross-treatment, including grazing (Graze) and enclosure management (Enclosure). The information obtained through investigation and interviews is that the enclosure and grazing age of the three enclosed grassland sample sites are more than 32 years. In our study, the enclosure treatment was considered as a control treatment for grazing grassland. we selected three representative grassland sites (locations) that met the predefined ecological criteria (e.g., vegetation type, soil characteristics, disturbance history). Within each 100 m × 100 m sampling area (an independent representative site), we randomly established five 1 m × 1 m quadrats for plant and soil sampling. The five quadrats within each 100 m × 100 m area were positioned to capture within-site heterogeneity while maintaining independence through minimum 20 m spacing between adjacent quadrats.

Because grazing grassland is not the kind of small-scale, precise sheep control experiment, but the grazing grassland is part of an open public rotational grazing pasture in winter and spring. Grazing intensity can only be estimated by interviewing herders. According to survey data from 15 to 18 households grazed at each sample site, the grazing intensity of the three sample sites were moderate to heavy grazing levels, as follows: the grazing intensity of the XL sample site is about 6.2-8.0 sheep units/ha/year. The grazing intensity levels of the EW samples ranged from 7.2 to 8.2 sheep units/ha/year, whereas the levels exhibited by the CB samples varied from 7.5 to 9.0 sheep units/ha/year.

### 2.3 Plant community survey

The experimental sample plots were assessed using a series of evenly distributed quadrats, measuring key indicators such as plant species composition, aboveground biomass (AGB), and belowground biomass (BGB). The coverage of grassland communities was measured using quadrat frame. Plant height was measured using a standardized five-point sampling method within each 1 m × 1 m quadrat. Five measurement points were uniformly distributed (center point and four intermediate points between center and corners). At each point, we measured the natural vertical height (without stretching) of the nearest dominant grass or forb species from the ground surface to the highest photosynthetic tissue using a rigid ruler (± 0.5 cm precision). The five measurements were then averaged to obtain the mean plant height for each quadrat. The survey recorded plant species and their abundance within each plot, classifying them into functional groups based on life forms: annuals or biennials (AB), perennial grasses (PG), perennial forbs (PF), and shrubs or subshrubs (SHS). For height measurement, five complete individuals of each functional group were randomly selected within each plot, and their vertical heights (from the base of the root collar to the tip of the highest leaf) in their natural growth state were measured using a graduated tape measure. The average value was taken after three repeated measurements for each individual. The total number of individuals and biomass for each functional group were also determined, followed by the calculation of relative abundance and relative biomass.

In each sample plot, plants were cut at ground level based on species classification, and dead plant material along with impurities were removed. Plant samples were collected, labeled according to species, and then transported to the laboratory for drying at 65°C for 48 hours until a constant weight was reached. The dried samples were then weighed to determine their AGB. Following AGB collection, three root samples were randomly extracted from each of the five plots using a 7 cm diameter soil corer, with sampling depths of 0–5 cm, 5–10 cm, and 10–20 cm. The collected soil samples were rinsed under running water, and the roots were separated using a 1 mm sieve, with dead roots and other residue removed. The root samples were then dried in an oven at 65°C for 48 hours until they reached a constant weight, and the BGB was determined by measuring their dry weight.

### 2.4 Determination of soil physical and chemical properties

Soil samples from the three layers (0–5 cm, 5–10 cm, and 10–20 cm) were collected using a 5 cm diameter soil coring drill from all five quadrats, with three cores of each layer combined to form a composite sample. The samples were then homogenized, sieved through a 2 mm mesh to remove stones and debris, and air-dried in preparation for subsequent physicochemical analyses. Soil organic carbon (SOC) content was measured using the dichromate oxidation method, while total nitrogen (TN) was quantified with an elemental analyzer (CHN vario MACRO cube, Germany). Total phosphorus (TP) was assessed using the acid digestion-molybdenum antimony blue colorimetric method. Microbial biomass carbon (MBC) and microbial biomass nitrogen (MBN) were determined via chloroform fumigation followed by K_2_SO_4_ extraction. Soil bulk density (BD) was assessed using stainless steel cores (5 cm diameter) collected from three soil profiles adjacent to randomly chosen quadrats. The microbial community structure was analyzed using phospholipid fatty acid (PLFA) profiling, and soil pH was recorded using a FE28 pH meter (Mettler Toledo, Switzerland).

### 2.5 Statistical analysis

We calculated the α-diversity index, which consists of two diversity indices, a richness index, and an evenness index.


(1) The Shannon−Wiener index:H'=−∑PilnPi

where P_i_ is the proportion of species individuals and i represents the relative density of plant species (number of species individuals/number of individuals of all species); N_i_ is the number of individuals of each species, P_i_=N_i_/N.


(2) $$\text{Simpson dominance index}:\text{D}=1-\sum {\text{P}}_{\text{i}}^{2}$$

(3) $$\text{Margalef richness index}:\text{R}=\left(\text{S}-1\right)/\text{lnN}$$

where S is the number of species and N is the number of all individuals.


(4) Pielou evenness index:E=H'/lnS

All data were processed using Excel 2016, analyzed with SPSS 24.0, and visualized in both Origin 2018 and Excel 2016. Before analyzing the data, the Kolmogorov-Smirnov (K-S) normal distribution test was conducted first. After the data passed this test, the T-test was used to directly compare the overall differences in plant diversity index (Shannon-Wiener index, Pielou evenness index, Simpson index, and Margalef richness index) and root shoot ratio between grazing and enclosure treatments. One-way ANOVA was used to analyze the differences among different plant biomass, soil microbial biomass, soil bacteria-fungal ratios, soil physicochemical properties, and plant functional group diversity under enclosure and grazing treatments. General linear model was used to analyze whether the influence of each factor on the dependent variable “soil properties” and the interaction effects among them were statistically significant. Linear model was used to examine the relationships between plant diversity and biomass (Simpson index with AGB and BGB), soil nutrients and plant biomass (SOC, TN, TP with TB, AGB, BGB), soil nutrients and root shoot ratio (SOC, TN, TP with root shoot ratio), and soil nutrients and plant diversity (SOC, TN, TP with Simpson index) under grazing and enclosure treatments. Structural equation modeling (SEM) was conducted via IBM Amos 24.0 to assess the interconnected effects of plant diversity, biomass, soil physicochemical properties, and soil nutrients under both grazing and enclosure conditions. In the SEM analysis, plant diversity (PD) is directly represented by the Simpson index. Microbial community structure (MCS) is dimensionally reduced via PCA analysis of the fungal to bacterial ratio (F:B), MBC and MBN. We retained MCS-PC1 (F:B ratio, with a total contribution rate of 76%) as the composite indicator. Soil nutrients (SN) are dimensionally reduced through PCA of SOC, TN, TP, and their stoichiometric ratios (C:N, C:P, N:P). We retained SN-PC1 (with a variance contribution rate of 91%, primarily driven by SOC and TN) as the composite indicator. Model fit is evaluated using the following indices: Chi-square degree of freedom ratio (χ^2^/df ≤ 3), comparative fitting index (CFI≥0.90), approximate root mean square error (RMSEA ≤ 0.10), goodness of fit index (GFI≥0.90), and gauge fitting index (NFI≥0.90) (MacCallum and Austin, 2000). The non-compliant models were optimized through theory-driven path adjustment (MacCallum and Austin, 2000).

## 3 Results and analyses

### 3.1 Effects of enclosure and grazing on plant diversity

**Figure 1:**
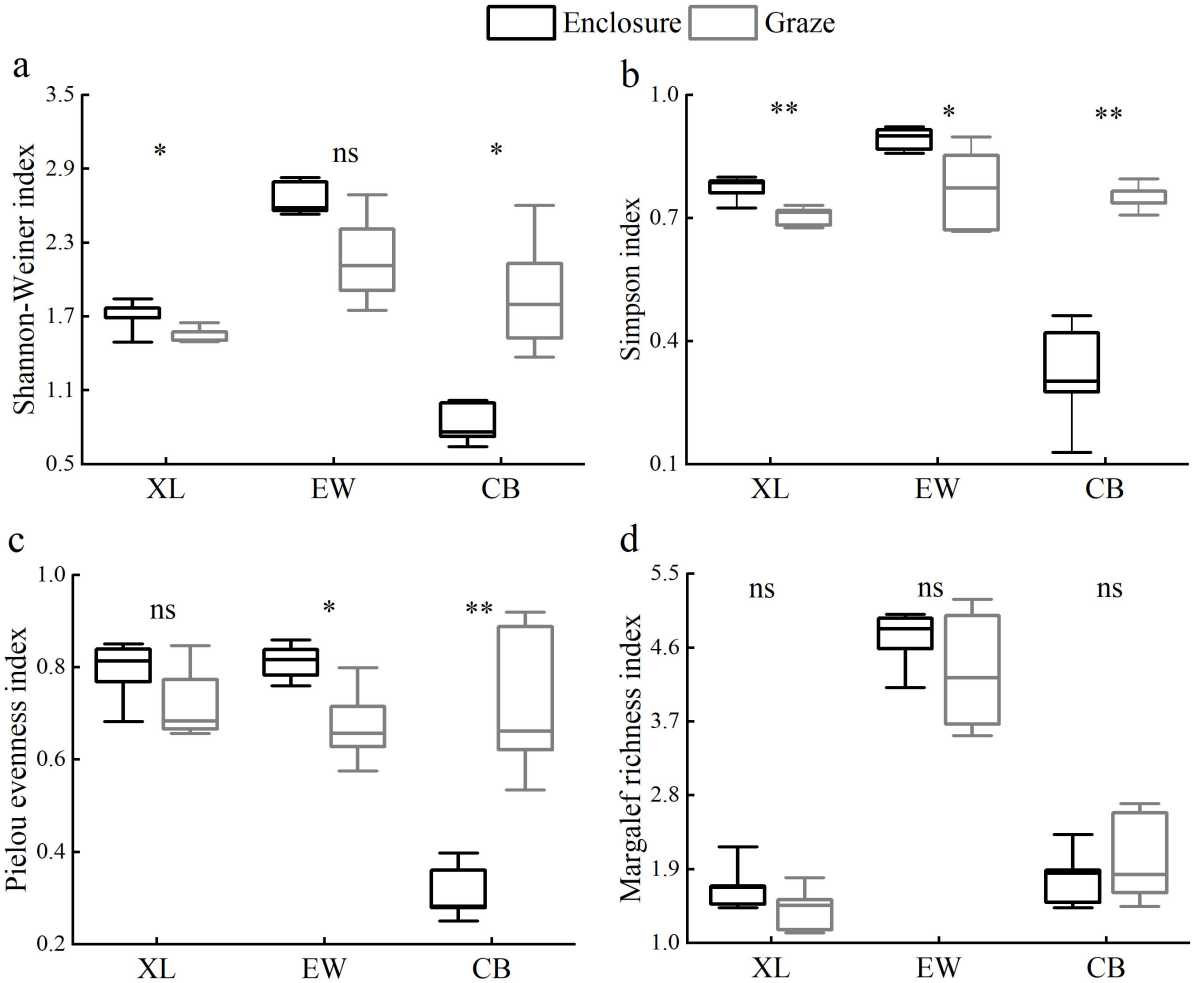
Effects of grazing and enclosure on plant species diversity indices, including Shannon-Weiner index **(a)**, Simpson index **(b)**, Pielou evenness index **(c)** and Margalef richness index **(d)**. "*" indicates a significant difference (P<0.05), "**" indicates a highly significant difference (P<0.01), and "ns" indicates a non-significant difference (P>0.05).

During the study period, the effects of grazing and enclosure on the Shannon-Wiener index, Pielou evenness index, Simpson index, and Margalef richness index (calculated by Equations 1–4) among the sample plots showed different results (**Figure 1**). The effects of enclosure and grazing on the Shannon-Weiner diversity index (*P*<0.05) and Simpson dominance index (*P*<0.01) of the XL sample plots were significantly different. There was no effect on the Pielou evenness index and the Margalef richness index, but the magnitude of the four diversity indices was enclosure>grazing for all four diversity indices. The effects of enclosure and grazing on the Pielou evenness index and Simpson dominance index of the EW sample plots differed significantly (*P*<0.05). They did not affect the Shannon-Weiner diversity index and the Margalef richness index, but the size of all four diversity indices was enclosure>grazing. Furthermore, the effects of enclosure and grazing on the diversity indices of CB sample plots were significant except for the Margalef richness index, and the size of the four diversity indices was enclosure<grazing.

### 3.2 Effects of enclosure and grazing on plant biomass and microbial biomass

**Figure 2:**
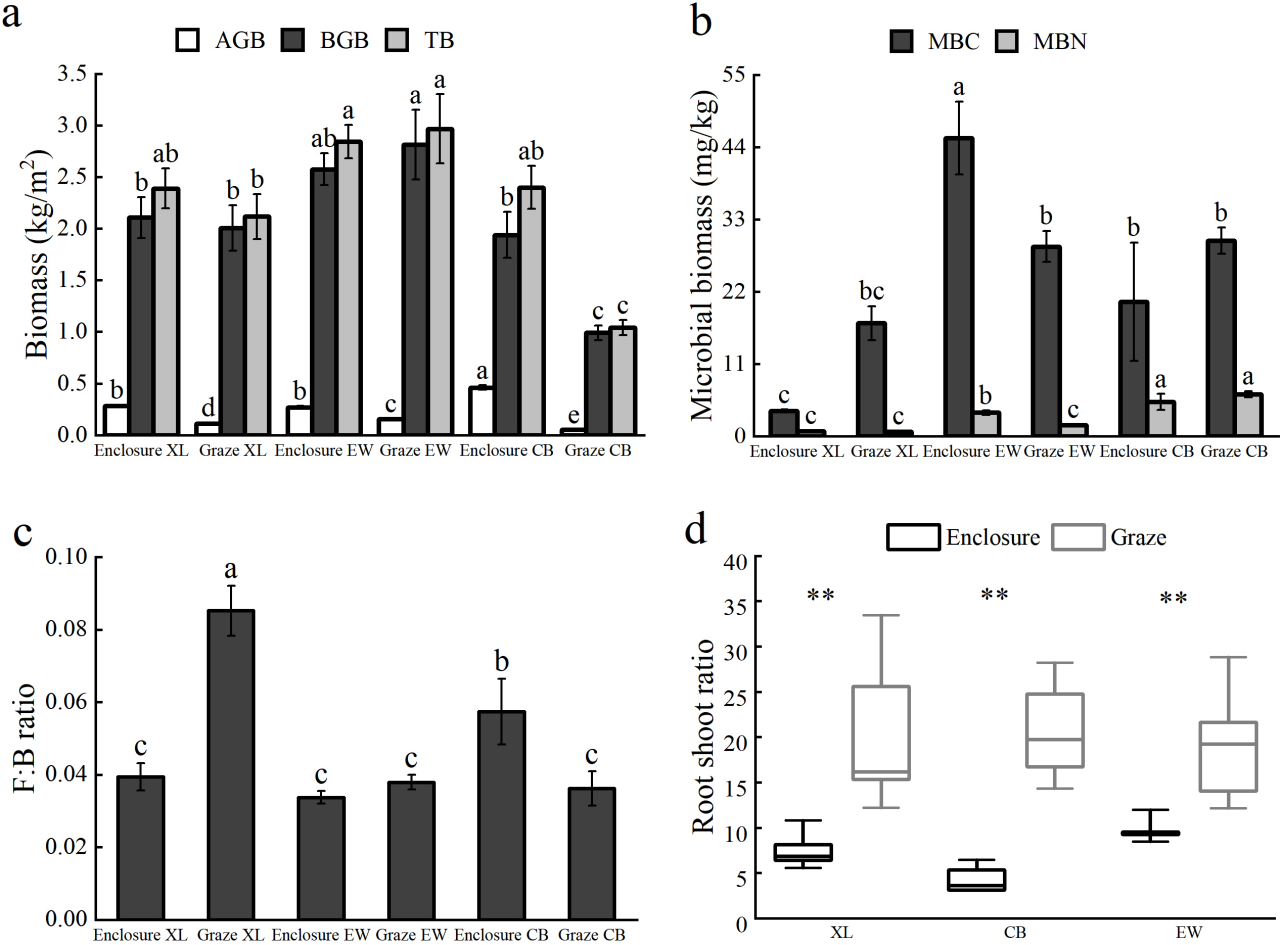
Characteristics of plant community biomass and soil microbial community in experimental site. **(a)**. Effects of enclosure and grazing on AGB, BGB, and TB; **(b)**. Effects of enclosure and grazing on microbial biomass of the community; **(c)**. Effects of enclosure and grazing on soil fungi-bacteria ratios; and **(d)** Effects of enclosure and grazing on root shoot ratio of the vegetation. Bar graphs represent means (error bars indicate Standard Error) (n=5), different lowercase letters represent differences between different sites (*P*<0.05), and “**” indicates highly significant differences between enclosure and grazing treatments (*P*<0.01).

Grazing significantly reduced AGB in all sample sites and BGB and total biomass (TB) in the CB sample plots but did not significantly affect BGB and TB in XL and EW, as compared to the enclosure treatment (**Figure 2a**).

Grazing did not significantly affect soil MBC and MBN in XL and CB but significantly reduced soil MBC and MBN in EW (**Figure 2b**). In addition, enclosure and grazing had significant effects on soil fungal-bacterial ratios (F:B ratios) except for sample site EW (**Figure 2c**), where enclosure decreased the fungal-bacterial ratio of XL, but increased the fungal-bacterial ratio of CB. Enclosure and grazing significantly affected the root-shoot ratio of vegetation in all three sample sites (**Figure 2d**), and the root-shoot ratios of the grazed grassland were significantly higher than those of the enclosure treatment (*P*<0.01).

**Figure 3:**
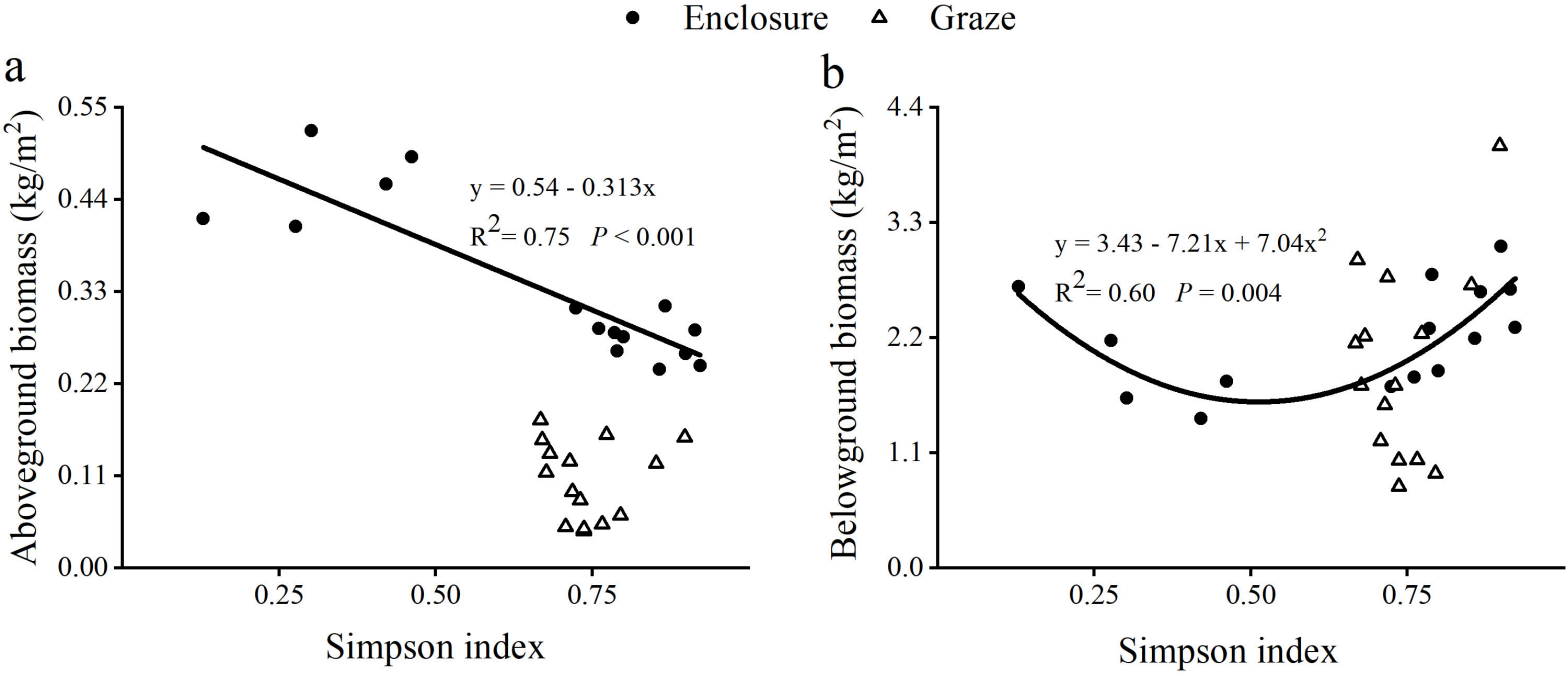
Regression analysis of community plant diversity and AGB **(a)** and BGB **(b)** under enclosure and grazing conditions.

In enclosed plots, Simpson’s diversity index exhibited a significant negative linear relationship with AGB (*P*<0.05), while showing a significant quadratic (U-shaped) relationship with BGB (*P*<0.05; **Figure 3**). The minimum BGB (1.59 kg/m^2^) occurred at a Simpson’s index of 0.51. No significant correlations were detected between plant diversity and either AGB or BGB in grazed plots (*P*>0.05).

### 3.3 Effects of enclosure and grazing on soil physicochemical properties and nutrients

**Figure 4:**
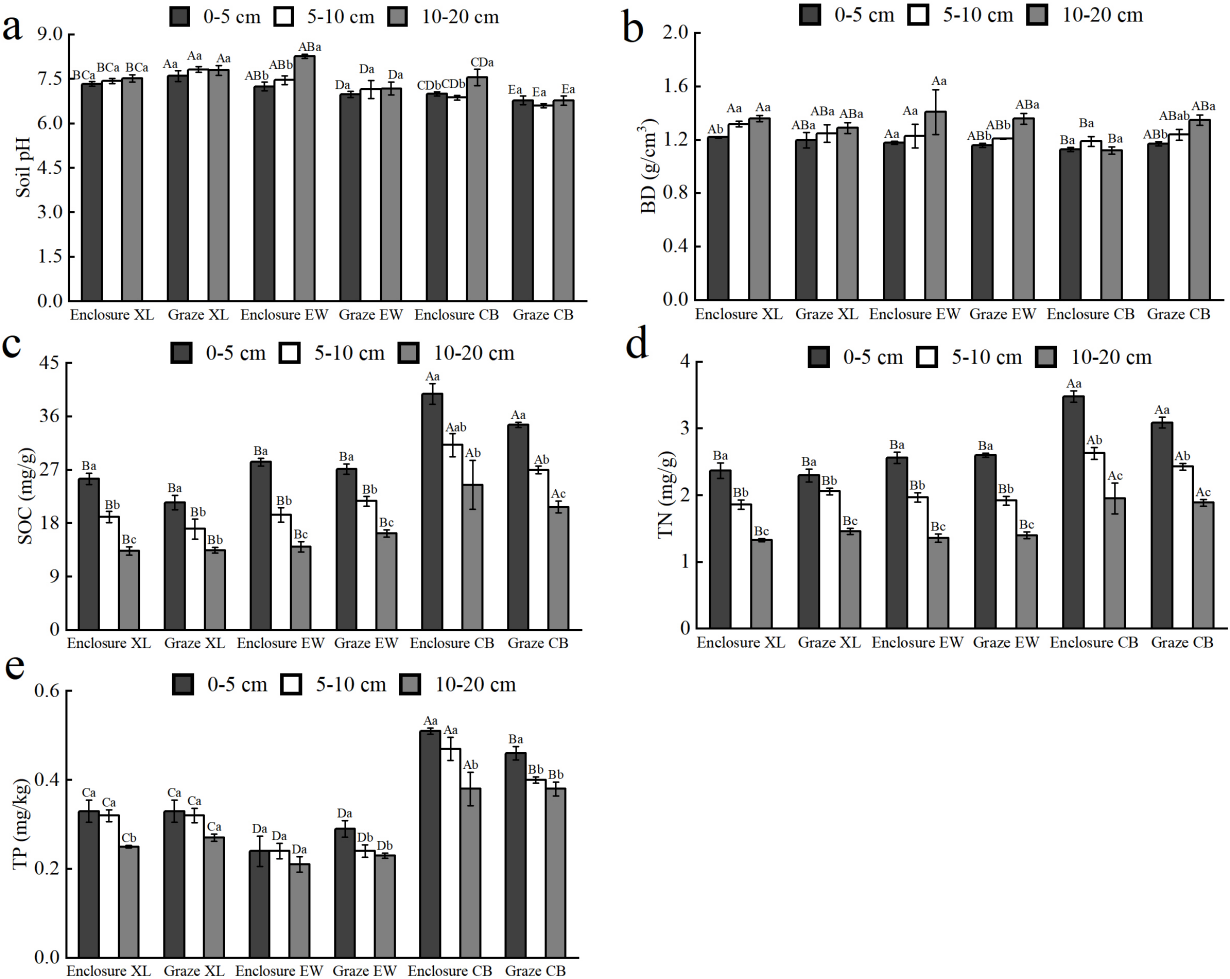
Soil physicochemical properties and nutrient content of three soil horizons in enclosed and grazed grassland, including soil pH **(a)**, soil bulk density (BD) **(b)**, soil organic carbon (SOC) **(c)**, soil total nitrogen (TN) **(d)**, and soil total phosphorus (TP) **(e)**. Each bar represents the mean (SE) (n=5) (except soil bulk density, n=3), lowercase letters represent variability among different soil horizons in the same site, and upper-case letters represent variability among different sites. Different letters represent significant differences (*P*<0.05).

The results showed (**Figures 4a, b**) that there was a significant difference (*P*<0.05) between the soil pH of grazed and enclosed grassland in all the sample sites; and there was no significant difference (*P*>0.05) in the bulk density (BD) of the soil between grazed and enclosed grassland. Soil temperature and pH decreased in grazed grassland compared to the enclosed treatment. Except for total phosphorus in CB plots, there were minimal differences in soil organic carbon, total nitrogen and total phosphorus between the plots under grazing and enclosure treatments. However, there were significant variations in SOC and TN across the three soil depth layers in different plots, with a decline observed with increasing the soil depth (*P*<0.05) (**Figures 4c–e**).

The present study conducted an analysis of ANOVA to explore the effects of Site, Soil depth, Grassland management, and their interaction effects on soil properties (**Table 2**). The results showed that for pH, Site (F=15.301, *P*<0.001), Soil depth (F=8.745, *P*<0.001), and Grassland management (F=15.976, *P*<0.001) all had extremely significant effects; significant interaction effects were also observed between Site and Soil depth (F=3.599, *P*<0.05), Site and Grassland management (F=4.028, *P*<0.05), and Soil depth and Grassland management (F=3.177, *P*<0.05). For BD, only Soil depth had an extremely significant effect (F=9.329, *P*<0.001), with no significant effects from other factors or interactions. SOC was significantly influenced by Site (F=82.82, *P*<0.001) and Soil depth (F=113.611, *P*<0.001), and the interaction between Site and Grassland management was also extremely significant (F=13.617, *P*<0.001). TN was extremely significantly affected by Site (F=80.564, *P*<0.001) and Soil depth (F=269.029, *P*<0.001), with Grassland management showing a significant effect (F=4.676, *P*<0.05) and the interaction of Site and Soil depth being significant (F=3.909, *P*<0.01). TP was extremely significantly influenced by Site (F=98.755, *P*<0.001) and Soil depth (F=20.648, *P*<0.001), and the interaction between Site and Grassland management was significant (F=4.388, *P*<0.05).

**Table 2:** The ANOVA results on the effects of different factors and their interaction effects on soil property indices.

Source	df	pH	BD	SOC	TN	TP
Site (S)	2	15.301***	2.185	82.82***	80.564***	98.755***
Soil depth (D)	2	8.745***	9.329***	113.611***	269.029***	20.648***
Grassland management (M)	2	15.976***	2.951	2.096	4.676*	2.016
S × D	4	3.599*	0.385	2.061	3.909**	0.683
S × M	1	4.028*	1.145	13.617***	1.389	4.388*
D × M	4	3.177*	0.703	0.997	0.528	0.619
S × D × M	2	0.101	0.783	0.155	2.212	1.275

***P<0.001, **P<0.01, *P<0.05, df represents degrees of freedom. The significance levels of the effects of different factors and their interaction effects on soil pH, BD, SOC, TN, and TP indices were indicated based on F-test results.

**Figure 5:**
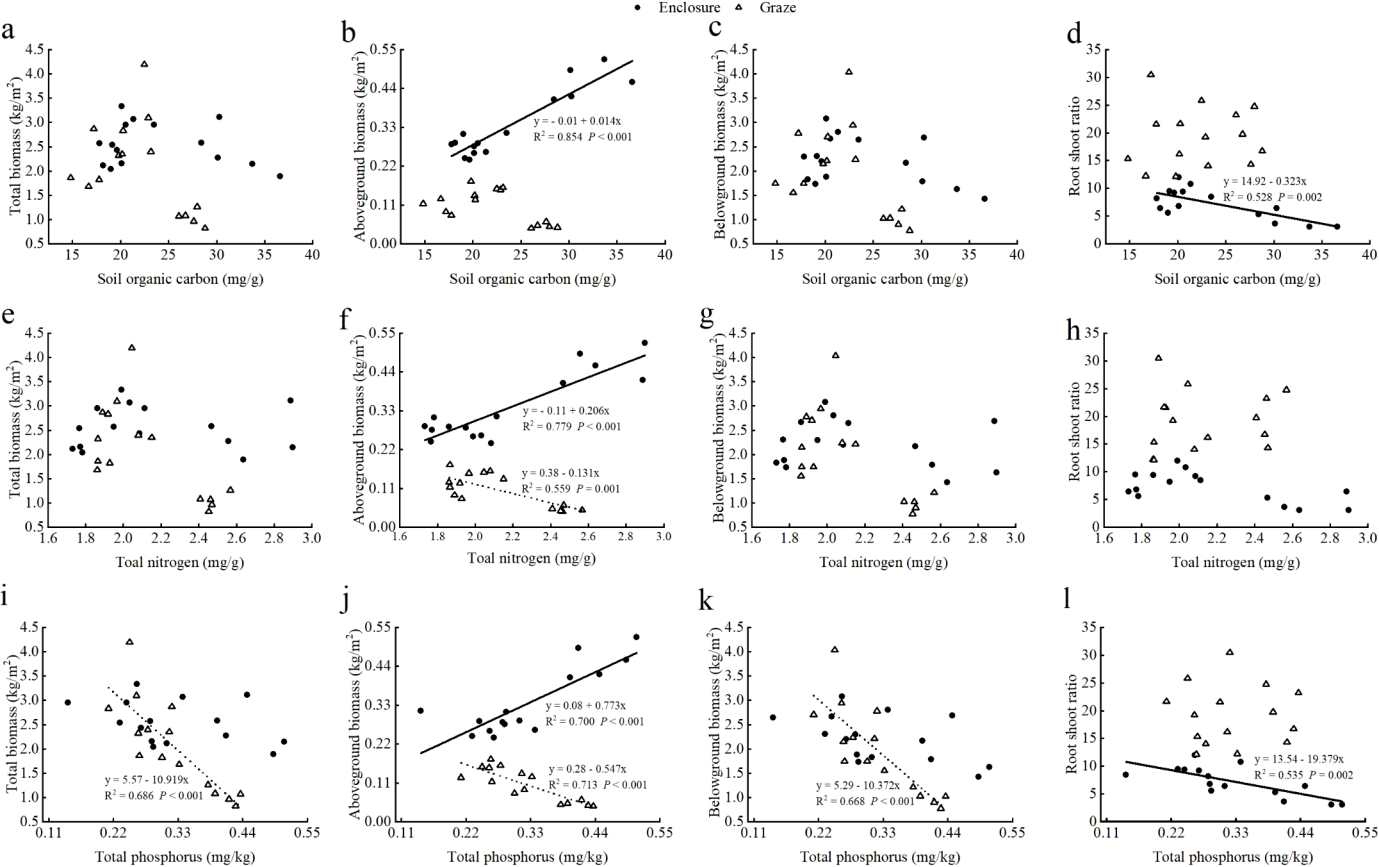
Linear regression analysis of the characteristics of plant biomass with soil nutrients, including soil organic carbon **(a-d)**, soil total nitrogen **(e-h)** and soil total phosphorus **(i-l)**, under enclosure and grazing conditions.

Regression analyses of plant biomass and soil nutrients showed (**Figure 5**) that enclosure increased plant AGB, which was positively linear regression with soil organic carbon, but decreased root shoot ratio, which was negatively linear regression with soil organic carbon, and had no significant effect on total biomass and BGB. Under grazing conditions, soil organic carbon had no significant effect on AGB, BGB, and TB, nor with root shoot ratio (**Figures 5a–d**). Total nitrogen was significantly related to AGB under enclosure and grazing conditions; enclosure increased AGB, but grazing decreased AGB, and total nitrogen had no significant effect on BGB, TB, and root-shoot ratio (**Figures 5e–h**). Total phosphorus significantly affected AGB, BGB, TB, and root shoot ratio (**Figures 5i–l**). Under grazing conditions, total phosphorus was negatively linear regression with AGB, BGB, and TB, with no significant effect on root shoot ratio, and under enclosure conditions, total phosphorus was positively linear regression with AGB, negatively linear regression with root shoot ratio, and had no significant effect on BGB and TB.

**Figure 6:**
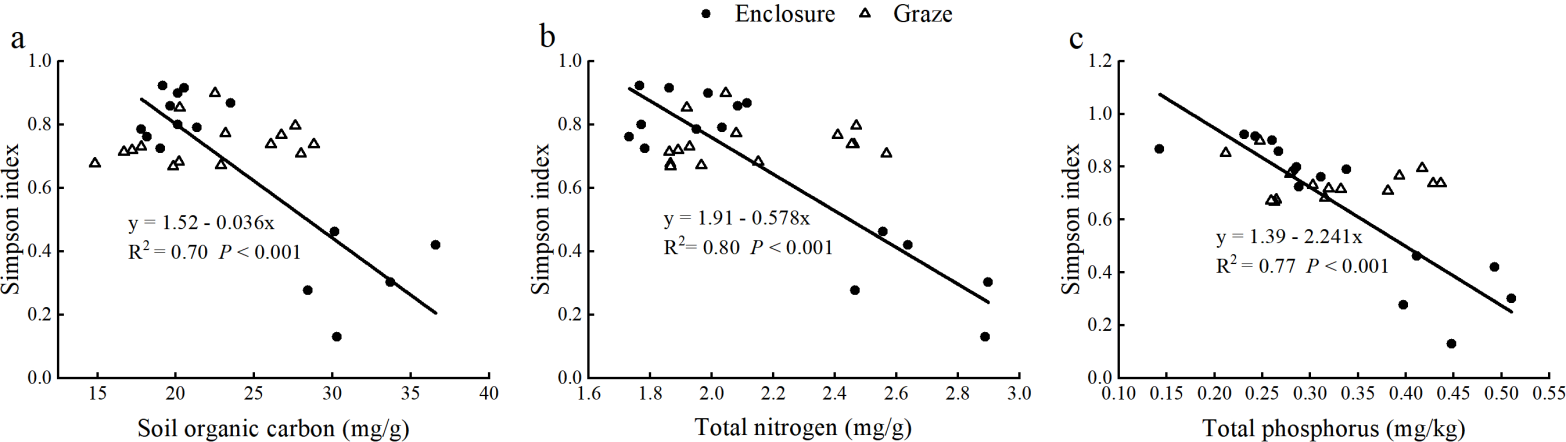
Linear regression analysis of soil nutrients, including soil organic carbon **(a)**, soil total nitrogen **(b)** and soil total phosphorus **(c)**, with plant species diversity under enclosure and grazing conditions.

The results of regression analysis showed that SOC, TN, and TP had a significant negative regression relationship with plant diversity under enclosure conditions (*P*<0.05) but no significant regression relationship under grazing conditions (*P*>0.05) (**Figure 6**).

### 3.4 Effects of enclosure and grazing on plant functional group diversity and biomass

**Figure 7:**
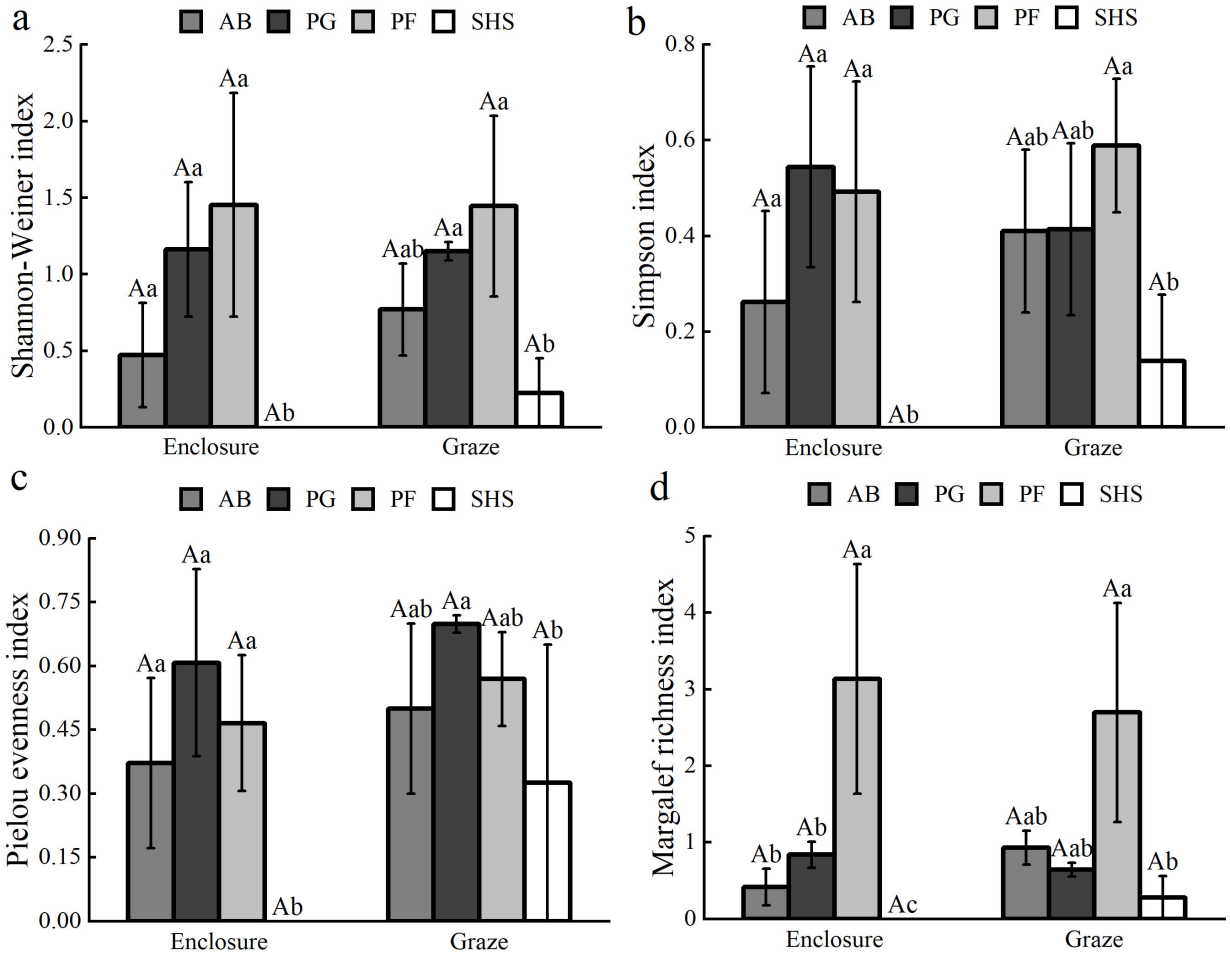
Effects of plant species diversity of each plant functional group, including Shannon-Weiner index **(a)**, Simpson index **(b)**, Pielou evenness index **(c)** and Margalef richness index **(d)**, in enclosed and grazed grasslands. Each bar represents the mean (SE) (n=3), different capital letters indicate differences in diversity of the same plant functional group between enclosure and grazing, and different lowercase letters indicate differences between different plant functional groups between the same treatments (*P*<0.05).

The species present in the community were categorized into four functional groups based on their life forms: AB, PG, PF, and SHS. As showed in **Figure 7**, the enclosure and grazing treatments did not significantly impact the Shannon-Wiener index, Simpson index, Pielou index, and Margalef index of the four functional groups, including AB, PG, PF, and SHS. However, under enclosed conditions, there was a significant difference in diversity index between the SHS functional group and other plant functional groups. Enclosure and grazing treatment did not significantly affect the Shannon-Wiener index, Simpson index, and Pielou index among AB, PG, and PF species. Furthermore, the Simpson index, Margalef index of PG, and Margalef index of PF were higher than those of grazing, whereas, for other plant functional groups, the diversity indices were higher than those of grazing.

**Figure 8:**
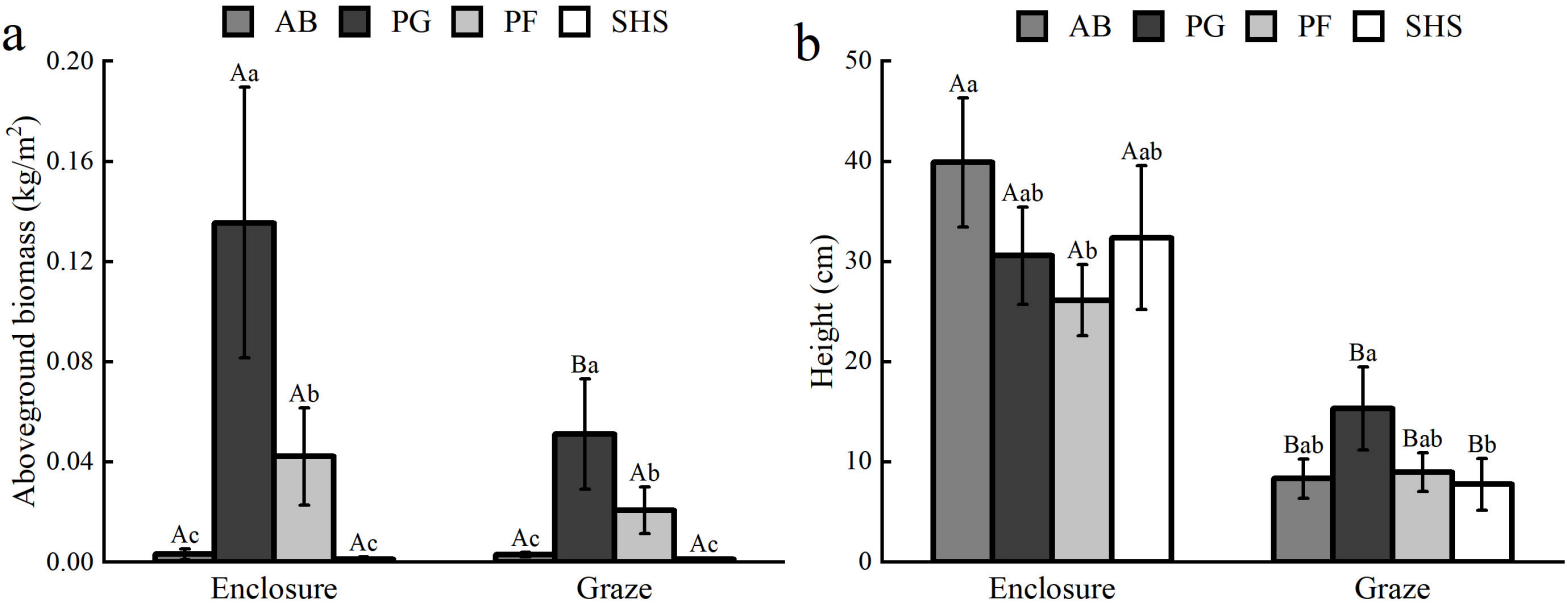
AGB **(a)** and plant height **(b)** of four functional groups of plants in enclosed and grazed grasslands. Each bar represents the mean value (error bars are the standard error) (n=3), with different capital letters indicating significant differences in the diversity of the same plant functional group between enclosure and grazing (*P*<0.05). Lowercase letters indicated significant differences between plant functional groups under the same enclosure or grazing treatment (*P*<0.05).

In terms of AGB of plant functional groups, only PG plant functional groups showed significant differences between enclosed and grazed grasslands (*P*<0.05) (**Figure 8a**). In the enclosed grassland, there were no significant differences in AGB between AB and SHS (*P*>0.05) and significant differences among other plant functional groups (*P*<0.05). The same pattern applies to grazing meadows. Compared with the grazing grassland, the PG functional group biomass of the enclosed grassland was 0.135 kg/m^2^, significantly higher than that of the grazing grassland (0.051 kg/m^2^). The biomass of the PG functional group was the highest in both grazing and enclosed grassland, followed by PF, AB, and SHS. In addition, the height of all plant functional groups in grazing grassland was significantly lower than that in enclosed grassland (*P*<0.05) (**Figure 8b**). There were significant differences in plant height between AB and PF in enclosed grassland (*P*<0.05). There were significant differences in plant height between PG and SHS in grazing grassland (*P*<0.05) but no significant differences in plant height among other functional groups (*P*>0.05).

### 3.5 Structural equation modeling path analysis of among factors under enclosure and grazing conditions

**Figure 9:**
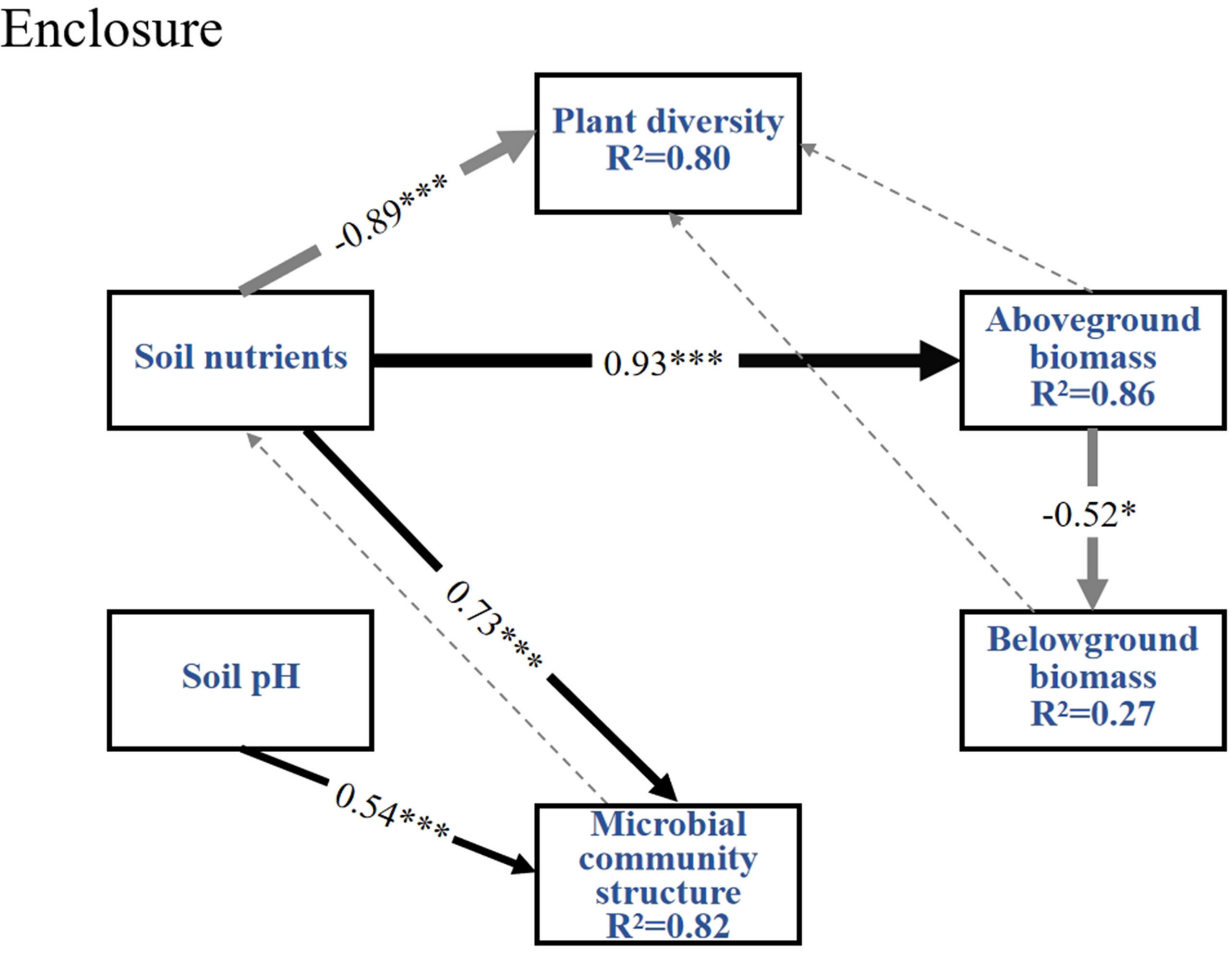
Structural equation modeling (SEM) was employed to analyze the interaction pathways between plant diversity, aboveground biomass (AGB), belowground biomass (BGB), microbial community composition, soil nutrients, and soil pH under enclosure conditions. Structural equation modeling under enclosure conditions with fit indices of Chi-square=20.239, d*f*=10, Chi-square/d*f*=2.024, *P*=0.027, GFI=0.765, AGFI=0.506, CFI=0.858, RMSEA=0.115; the widths of the solid arrows denote the significant standardized path coefficients as indicated by ***P<0.001, *P<0.05; black solid arrows represent path coefficients that are significantly positively linear regression, light grey solid arrows represent path coefficients that are significantly negatively linear regression, and grey and black dashed arrows represent non-significant paths.

The SEM explains 86% of the AGB variables, 82% of microbial community structure variables, 80% of plant diversity variables, and 27% of BGB variables under enclosure treatment (**Figure 9**). Results indicate that soil nutrients exert a significant negative effect on plant diversity [path coefficient (β)=-0.89], but positively promote AGB (β=0.93) and microbial community structure (β=0.73). Additionally, soil pH positively drives microbial community structure (β=0.54). Notably, AGB and BGB exhibit a significant negative linear regression relationship (β=-0.52), reflecting a trade off in resource allocation between aboveground and belowground components.

**Figure 10:**
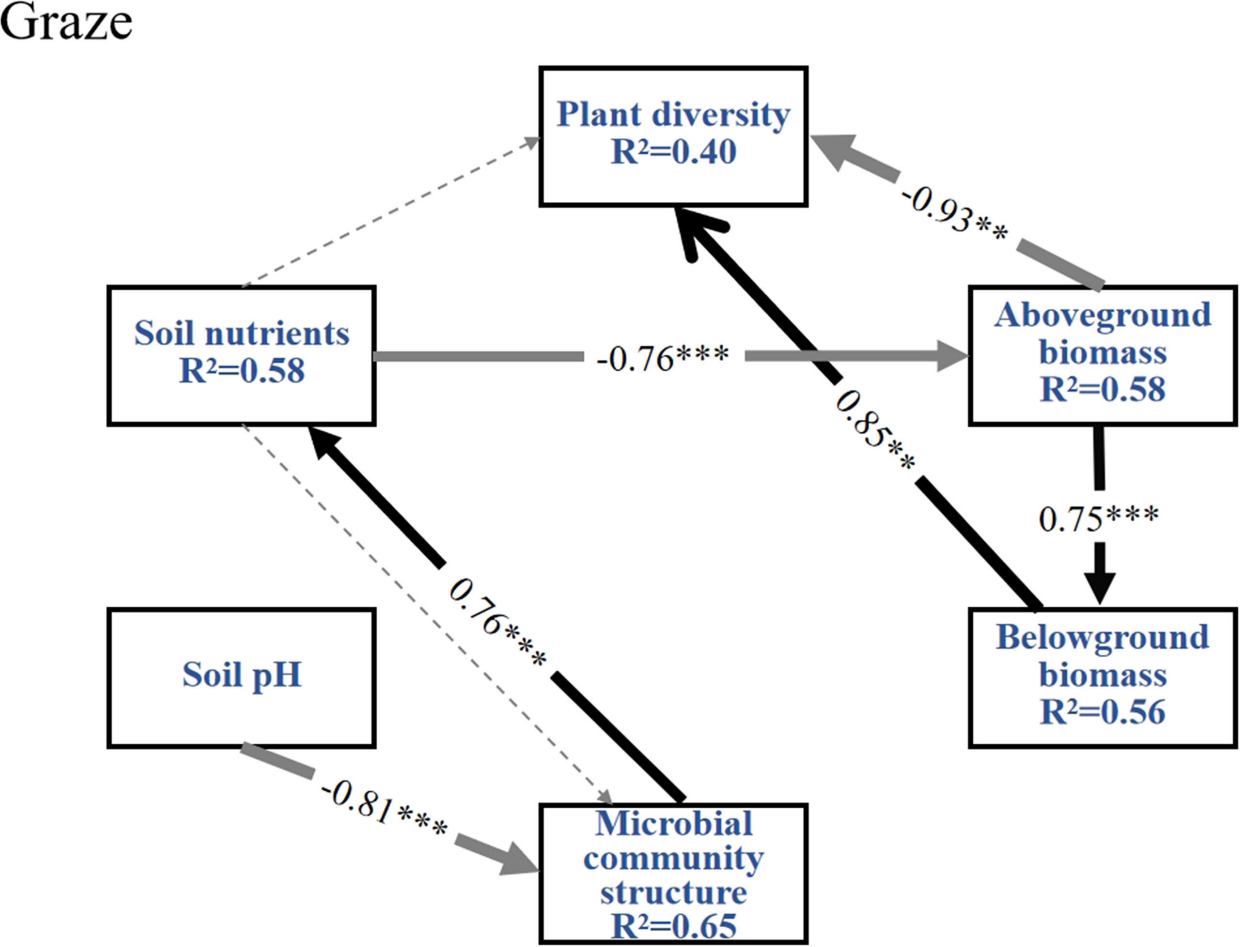
Structural equation modeling (SEM) was employed to analyze the interaction pathways between plant diversity, aboveground biomass (AGB), belowground biomass (BGB), microbial community composition, soil nutrients, and soil pH under grazing conditions. Structural equation modeling under grazing conditions with fit indices of Chi-square=7.675, d*f*=9, Chi-square/d*f*=0.853, *P*=0.567, GFI=0.873, AGFI=0.704, CFI=1.000, RMSEA=0.000; the widths of the solid arrows denote the significant standardized path coefficients as indicated by ***P<0.001, **P<0.01; black solid arrows represent path coefficients that are significantly positively linear regression, light grey solid arrows represent path coefficients that are significantly negatively linear regression, and grey and black dashed arrows represent non-significant paths.

The SEM explained 65% of the microbial community structure variables, 58% of the soil nutrient variables, 58% of the AGB variables, 56% of the BGB variables and 40% of the plant diversity variables under grazing treatment (**Figure 10**). The results show that: Soil nutrients negatively affect AGB (β=-0.76), while microbial community structure positively drives soil nutrient accumulation (β=0.76). Soil pH negatively impacts microbial community structure (β=-0.81). Additionally, AGB significantly inhibits plant diversity (β=-0.93), but AGB positively promotes BGB (β=0.75), Meanwhile, BGB significantly enhanced plant diversity (β=0.85), reflecting a compensatory mechanism in biomass allocation under grazing disturbance.

## 4 Discussion

### 4.1 Characterization of plant diversity in response to grazing and enclosure grasslands

Plant community biodiversity plays a crucial role in maintaining grassland ecosystem balance and productivity (Tilman et al., 1996). Generally, grazing tends to reduce biodiversity, whereas enclosure over time increases both plant abundance and biodiversity (Filazzola et al., 2020). In low-stress ecosystems, however, grazing may reduce its impact on plant communities by stimulating compensatory plant growth (Ramula et al., 2019). In this study, plant diversity was higher in the XL and EW samples under the enclosure treatment. However, plant diversity in the CB sample showed the opposite trend, with grazing increasing diversity (**Figure 1**). This might be due to overgrazing, which leads livestock to prioritize the consumption of herbaceous plants with strong palatability, reducing their competitive edge. As a result, shrubs with strong grazing tolerance obtain more resources and expand their ecological niches. This niche differentiation increases species diversity by altering the structure of plant communities (Shi et al., 2023). The long-term heavy grazing at CB likely suppressed dominance by competitive perennial grasses, creating microsites for subordinate species establishment. This aligns with disturbance-mediated coexistence theory, where intense grazing disrupts competitive hierarchies, allowing annuals and smaller shrubs to thrive (Zhang et al., 2023a).

In addition, the inconsistent pattern of plant diversity change in the CB grazing samples may be attributed to variations in precipitation between sampling sites (Zhao et al., 2011). Precipitation influences how livestock grazing affects plant diversity (Herrero-Jáuregui and Oesterheld, 2018). Grazed grasslands typically exhibit higher species richness in wetter, more productive ecosystems (Lezama et al., 2014). Both the XL and EW sites belong to the semi-arid continental climate of the middle temperate zone, with less precipitation (290–300 mm) and lower productivity. Under grazing treatment, the living environment of plants is damaged, resulting in a decrease in species diversity. However, enclosure creates conditions for plant growth and is conducive to vegetation restoration efforts (Miao et al., 2015). Therefore, the diversity of enclosed plants in semi-arid areas is greater than that of grazing. However, the CB site belongs to the temperate continental climate, with more precipitation (320 mm). In wetter and more productive ecosystems, grazing plots typically have higher species richness (Lezama et al., 2014), so species diversity is higher under grazing conditions at the CB site. Several studies in southern Africa have shown significant increases in species diversity under heavy grazing in low-productivity systems with a long history of grazing (Hanke et al., 2014). The CB site’s unique phenomenon reflects a balance where heavy grazing overrides precipitation-driven competitive dominance. In contrast, at XL/EW, enclosure combined with insufficient rainfall reduced perennial grass monopolization, promoting diversity. These patterns reached an agreement with studies showing that grazing effects on diversity depend on both disturbance intensity and environmental context (Zhang et al., 2023a).

Additionally, the interaction between grazing and abiotic stresses can have complex effects on plant diversity. Typically, increasing grazing intensity and animal trampling alter the spatial distribution of soil pores, leading to higher soil infiltration resistance and increased bulk density, which reduces soil water retention and affects plant diversity (Herrero-Jáuregui and Oesterheld, 2018). However, no significant difference in soil bulk density was observed between enclosure and grazing treatments in this study (**Figure 4c**), which could be due to environmental factors having a stronger influence on soil properties than grazing. The effect of grazing on soil bulk density may vary depending on soil moisture content and texture (Rakkar and Blanco-Canqui, 2018).

Under enclosure conditions, soil nutrients had a significant negative impact on plant diversity, which declined with increasing levels of soil organic carbon, total nitrogen, and total phosphorus. In contrast, no significant relationship was observed between soil nutrients and plant diversity following grazing treatments (**Figure 6**). This may be due to reduced nutrient return to the soil from grazing-induced decomposition of plant litter, leading to an imbalance between soil nutrients and vegetation characteristics (Klumpp et al., 2009). Therefore, under grazing conditions, plant diversity is not regulated by soil nutrients. Grazing can either increase or decrease plant diversity, depending on the type and intensity of grazing, as well as the climatic conditions of the site (Filazzola et al., 2020). In this study, all grazing sample sites were free-grazing grasslands, indicating that grazing animals may be a key factor influencing plant diversity. For example, sheep exhibited higher dietary selectivity than cattle. Grasslands grazed by sheep had lower plant diversity, functional diversity, and grass coverage compared to those grazed by cattle (Tóth et al., 2016).

We investigated the relationship between plant functional groups and environmental factors by considering all species (Li et al., 2021b). Plants adapt to variable environments by reorganizing into different functional groups to thrive in heterogeneous habitats (Petchey and Gaston, 2002). This study found that enclosure and grazing treatments did not significantly impact the diversity indices of plant functional groups. However, all four diversity indices for AB and SHS showed higher values under grazing than under enclosure (**Figure 7**). This may be because perennial grasses, such as *Leymus chinensis*, dominate the upper layers of the community and are more palatable. As grazing intensity increases, their dominance and interspecific competitiveness decline. This leads to a contraction in their ecological niches (Jing et al., 2017), allowing the niches of annual and biennial grasses and shrubs to expand, thereby increasing their proportion in the AB and SHS communities. This also highlights the growth trade-offs among different plant functional groups within the community in response to external disturbances. It is important to note that management response trends in plant functional group diversity require further validation through expanded sample sizes. Current results merely indicate potential directional effects, not definitive conclusions.

### 4.2 Comparison of plant biomass under enclosure and grazing conditions

In our study, the grassland AGB of plants in the enclosure samples was significantly higher than in the grazing samples. However, the root-to-shoot ratio was significantly lower in the enclosure samples than in the grazing samples (**Figures 2a, d**). Repeated trampling and foraging by livestock on surface vegetation led to a reduction in aboveground vegetation cover (Herrero-Jáuregui and Oesterheld, 2018). Grazing can lead to a significant loss of leaves, thereby inhibiting photosynthesis and reducing the compensatory growth of AGB, ultimately resulting in a significant decrease in AGB in grassland (Liu et al., 2023). At the same time, grazing alters the allocation of matter and energy among various organs of forage grass, generally promoting the distribution of belowground biomass, thereby increasing the root-shoot ratio of plants (Pucheta et al., 2008). Our study revealed a negative correlation between AGB and plant diversity under enclosure conditions. In contrast, no significant relationship was observed under grazing conditions (**Figure 3a**), which may be due to inconsistent plant diversity levels among the sample plots. At low plant diversity levels, plant AGB decreased, and the differences between grazing treatments were insignificant, whereas grazing at high plant diversity levels increased plant AGB (Bai et al., 2015).

Grazing intensity is a crucial factor influencing plant biomass in grasslands. Light grazing can stimulate plant overcompensation for growth, thereby increasing the AGB of grasslands, whereas heavy grazing significantly reduces plant AGB (Bai et al., 2015). Heavy grazing significantly reduces BGB in temperate grasslands, whereas moderate grazing significantly increases BGB (López-Mársico et al., 2015). In our study, although the differences in BGB between enclosure and grazing treatments in XL and EW plots were not statistically significant, the mean BGB of enclosure in XL and CB plots was higher than that of grazing, and the mean BGB of enclosure in EW plots was lower than that of grazing. Moreover, the changing trends of BGB and MBC under enclosure and grazing treatments in the three plots were opposite (**Figures 2a, c**). This indicates that soil microbial biomass carbon may be related to the content of BGB. This might be because MBC affects the availability of nutrients by regulating the mineralization and fixation processes of soil nutrients, thereby exerting a negative effect on the growth of BGB (Jiang et al., 2024a).

Changes in plant diversity under enclosure treatments also influenced BGB. As Simpson’s index increased, a U-shaped regression pattern was observed between BGB and the grassland diversity index (**Figure 3b**). The inflection points of the lowest BGB value can therefore be utilized to enhance root productivity within the community. In our study, when the BGB of enclosed grassland exceeded a certain threshold (1.5-1.7 kg/m^2^), we found that the community exhibited two growth strategies: either increasing or decreasing plant diversity could enhance BGB. Furthermore, grazing significantly reduced the height of the four plant functional groups compared to enclosure, but only decreased the AGB of the PG functional group, with no significant effect on the AGB of the other functional groups (**Figure 8**). This could be attributed to the ecosystem’s resilience. Besides livestock’s preference for forage PG, the three plant functional groups AB, PF, and SHS exhibited compensatory growth under moderate grazing conditions (Ramula et al., 2019).

### 4.3 Potential links between soil physicochemical properties and microbial community structure in grazed and enclosed grasslands

Soil microbial communities regulate nutrient cycling processes and plant productivity, serving as important indicators of soil health (Fan et al., 2020). In our study, soil and microbial community characteristics exhibited greater complexity between enclosed and grazed grasslands. Compared with enclosure, grazing activities had no significant effect on MBC and MBN in the XL and CB plots, but significantly reduced MBC and MBN in the EW plot (**Figure 2b**). The enclosure and grazing treatment of the EW plot had no significant effect on the F:B ratio. The enclosure of the XL plot reduced the F:B ratio, while that of the CB plot increased it (**Figure 2c**). Overall, in the three plots, the responses of MBC, MBN and F:B ratio to enclosure and grazing presented a complex variation pattern. This may be attributed to the complexity of the sample site’s environmental and microbial networks, as well as the effects of enclosure and grazing on biomass and structure. Both moderate grazing and enclosure contributed to increased soil MBC, as moderate grazing enhances root exudates, stimulating microbial activity (Holland et al., 1996). Grassland type and microbial abundance determine the amount and ecological distribution of soil MBN (Joergensen et al., 1995). Soil microbial abundance and activity were influenced by nutrient status, texture, vegetation composition, and cover (Díaz-Raviña et al., 1995). At large regional scales, microbial coexistence networks are primarily influenced by average annual rainfall, while at smaller scales, they are mainly shaped by local soil and plant factors (Wang et al., 2018).

In this study, SOC and TN exhibited a decreasing trend under grazing compared to enclosure (**Figures 4c, d**), likely due to the removal of surface biomass by grazing. This reduction limited nutrient replenishment (Jiang et al., 2024b). In the CB sample plots, TP significantly increased under enclosure compared to grazing (**Figure 4e**). This might be attributed to grazing increasing phosphorus export rates and reducing soil phosphorus levels. In contrast, enclosure prevented livestock foraging and reduced nutrient and energy export from the ecosystem, thereby increasing soil TP content (Wu et al., 2009). Additionally, the vertical distribution characteristics of soil nutrients (e.g., SOC and TN) were significantly different (*P*<0.05) (**Figure 4**; **Table 2**). These patterns suggested management practices differentially redistribute resources vertically, altering plant competition strategies. Dominant perennial grasses (e.g., *Leymus chinensis*) in enclosures exploited surface nutrients, suppressing shallow-rooted annuals through light competition in the shallow roots (0–20 cm) (Shi et al., 2025).

Soil pH directly influenced the efficiency of nutrient uptake by grassland vegetation. Acidification can significantly alter the structural diversity of microbial communities in grazed grasslands (Martins et al., 2020). In this study, the pH of each soil layer under grazing treatment in the XL plot was significantly higher than that under enclosure treatment, but the soil pH of each soil layer under grazing treatment in the EW and CB plots was lower than that under enclosure treatment (**Figure 4a**). This could be attributed to the increase in livestock urine deposition as grazing intensity rises. Increased livestock urine accelerated soil ion cycling, elevating soil hydrogen ion concentrations and thus lowering soil pH (Woodbridge et al., 2014). However, in the XL plot, the pH of each soil layer under grazing treatment was significantly higher than that under enclosure treatment. This might be because the grazing intensity in the XL plot was more appropriate. Moderate grazing can increase soil porosity, improve soil moisture conditions, and thereby increase soil pH (Lai and Kumar, 2020). Additionally, in the EW and CB sites, the pH decline magnitude in the 10–20 cm soil layer was greater than in the 0–5 cm and 5–10 cm layers. This may be attributed to grazing altering plant root distribution and promoting the growth of deep-rooted plants. Secretions from deep roots (e.g., organic acids) or acidic substances derived from decomposed dead roots could accumulate in the 10–20 cm soil layer, directly lowering the pH (Panchal et al., 2021). Furthermore, the absorption of basic ions by the root system may intensify the depletion of basic ions in deep soil and weaken its antioxidant capacity (Lai and Kumar, 2020).

### 4.4 Linkages between plant diversity and ecosystem functional properties in enclosed and grazed grasslands

Understanding the relationships between elements in grassland ecosystems and their driving pathways is essential for a thorough comprehension of their structure and function (Demmer et al., 2018). In the enclosure treatment (**Figure 9**), soil nutrients exhibit a significant negative effect on plant diversity, which may be attributed to intensified interspecific competition among plants driven by available nutrient of soil (Li et al., 2017). In enclosed grassland, soil nutrients enhance AGB and microbial communities by improving resource availability, thereby boosting the productivity of dominant perennial grass species (Du et al., 2025). The negative effect soil nutrients on the AGB under grazing (**Figure 10**) probably reflected nutrient depletion and microbial immobilization rather than direct inhibition. Long-term grazing accelerated N/P leaching through soil compaction and reduced vegetation cover (Li et al., 2022). And the soil microbial communities were shifted toward oligotrophic taxa that immobilize nutrients in microbial biomass (Wang et al., 2020). These processes created a nutrient-poor rhizosphere, forcing plants to prioritize root proliferation over shoot growth, thereby suppressing AGB despite residual soil nutrient levels. This aligned with observed root-shoot ratio increases and the regression relationship between soil nutrients and AGB under grazing grasslands (**Figures 2d**, **5**).

The significant negative linear regression relationship between AGB and BGB in enclosures (**Figure 9**) might indicate that plants prioritize resource allocation to aboveground parts to enhance photosynthetic capacity under undisturbed conditions (Bricca et al., 2023). In contrast, under grazing treatment (**Figure 2a**), grazing dominates the dynamics of AGB through direct trampling and selective feeding removal by herbivores, significantly restricting grassland productivity. The negative correlation between AGB and plant diversity under grazing pressure **Figure 10**) may result from the dominance of grazing resistant functional plant types, exacerbating resource competition (May et al., 2009). Additionally, in contrast to enclosure treatment, the positive effect of AGB on BGB under grazing conditions may reflect a compensatory strategy where plants increase belowground resource storage to cope with livestock stress (Bricca et al., 2023). This strategy reduced competition for light resources, allowing low-growing or trampling-tolerant species to occupy niches within the community, thereby enhancing plant diversity through BGB (Hao and He, 2019).

Additionally, a significant negative linear regression of AGB with plant diversity and a significant positive linear regression with soil nutrients were observed under enclosure treatment (**Figures 3a**, **5**, **9**). After the enclosure, the physicochemical properties of the grassland soil were significantly improved, such as the soil aeration and moisture storage capacity increased (Jing et al., 2017). Improved moisture content enhances nitrogen utilization efficiency (Wang et al., 2020). The improvement of soil nutrients, especially available nitrogen, would shift the plant community from multiple species to sole dominance by *Leymus chinensis* or *Stipa* spp (Zhang et al., 2025). In this study, AGB significantly reduced species richness under enclosure conditions (**Figure 3a**), possibly due to the increased community productivity from enclosure treatment, particularly the AGB of grasses and forbs. This reduced community gaps and the plant litter cover effect, leading to the loss of species less competitive for light resources (Semmartin et al., 2008). The direct negative effect of enclosure on plant diversity and soil nutrients may be linked to root nutrient uptake dynamics. This provides a deeper understanding of how plant productivity influences species diversity and the accumulation of soil elements in typical grassland ecosystems.

Soil microbes are important drivers of plant diversity and productivity in grasslands (Schnitzer et al., 2011). By regulating the function of soil microorganisms, it is possible to link the cycling processes of plant and soil nutrients (Schimel and Schaeffer, 2012). As a result, soil nutrients influenced microbial populations and community structure, which were higher under enclosure conditions (**Figure 4**). However, in most studies, soil microorganisms may be primarily limited by phosphorus, likely due to the generally low concentration of readily available phosphorus, poor mobility, and high heterogeneity (Čapek et al., 2018). Thus, soil nutrients under enclosure conditions have a highly significant direct positive effect on microbial community structure (**Figure 9**). Phosphorus addition indirectly affects microorganisms, primarily by influencing the soil carbon cycle and chemical properties (Huang et al., 2016). Under grazing treatment (**Figure 10**), the promotional role of microbial activity on soil nutrient cycling might stem from accelerated litter fragmentation due to trampling by grazing animals, which enhanced microbial decomposition efficiency and facilitates nutrient release (Wei et al., 2021; Du et al., 2025). However, the significant inhibition of microbial community structure by soil pH indicated that grazing might indirectly alter material cycling processes by modifying microenvironmental pH (Liu et al., 2024). A key element that influences the variety and composition of soil bacterial and fungal groups was the pH of the soil (Liu et al., 2018). According to this study, compared to enclosure, grazing disturbance led to a direct negative effect of soil pH on microbial community structure (**Figures 9**, **10**). Lower soil pH in the management of grazing could be the cause of this change because it would reduce the amount of organic carbon that decomposes in the soil. Furthermore, because of the biotoxicity of exchangeable aluminum ions in soil solution, lower pH decreased the amount of carbon that is transferred from plants to soil and bacteria (Pietri and Brookes, 2008). While enclosure stabilized soil properties and pH by reducing livestock disturbance, allowing microbial communities to adapt and form stable structures aligned with prevailing pH conditions.

## 5 Conclusions

(1) The enclosure reshaped the perennial plant communities to promote plant growth in terms of height and biomass. This counteracted the advantages of annual species under grazing by promoting stable perennial grasses.
(2) Under enclosure conditions, the asynchrony between AGB and plant species diversity is the result of separate regulation by soil nutrients. However, under grazing conditions, the reduction of AGB may lead to decreased plant diversity; but this process is partially offset by the modulatory effect of BGB.
(3) Grazing altered the relationship between soil nutrients and plant biomass. The impact of grazing on AGB was severe, but it also transformed the relationship between AGB and BGB from competitive restrictions under enclosure to synergy in grazing.
(4) Under enclosure treatment, soil nutrients and pH values regulated the microbial community via a direct positive effect pathway. Under grazing interference, soil pH affects soil nutrients by regulating the structure of the microbial community. A comparison of the two grassland management types revealed that the interaction between soil nutrients and microorganisms exhibits reversed patterns.

## Data Availability

The original contributions presented in the study are included in the article/**Supplementary Material**. Further inquiries can be directed to the corresponding authors.
