# Supplementary material for: Soil function, properties and plant diversity–biomass patterns differ between grazed and non-grazed steppe ecosystems
Source: Front Plant Sci. 2025 Jul 8;16:1597590. doi: 10.3389/fpls.2025.1597590 (PMC12280993; doi:10.3389/fpls.2025.1597590)
Supplement: Supplementary file 2 [file DataSheet1.docx]

**Supplementary Material**
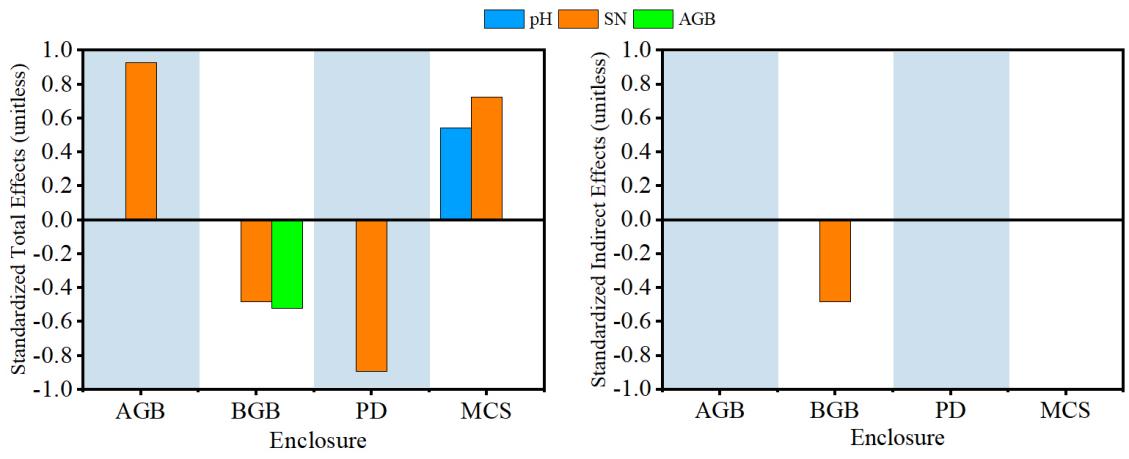


Fig. S1. The standardised total and indirect effects between ecological factors under enclosure conditions

Notes: pH: soil pH; MCS: microbial community structure, which is a dimensionality reduction value of F:B, MBC and MBN; AGB: aboveground biomass; BGB: belowground biomass; PD: plant diversity; SN: soil nutrients, which is a dimensionality reduction value of SOC, TN, TP, C:N, C:P and N:P.


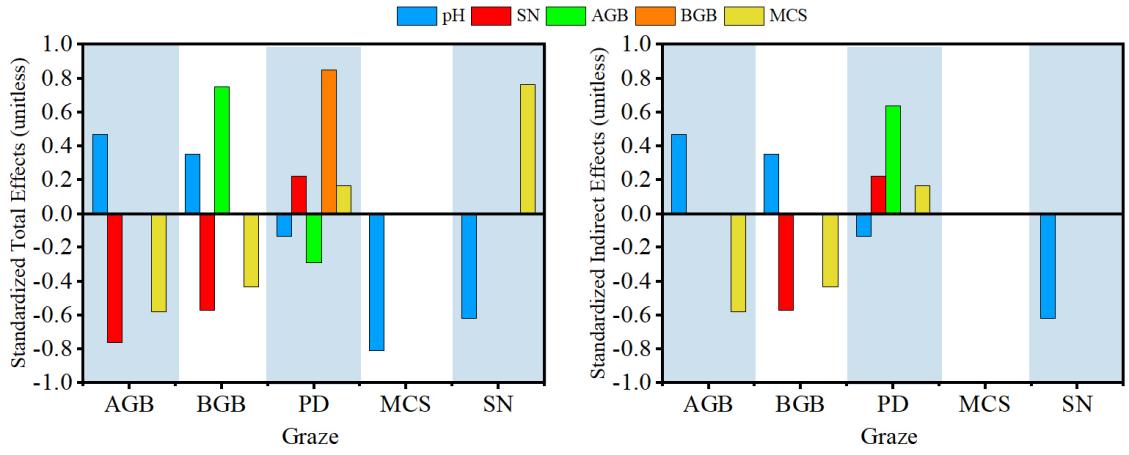


Fig. S2. The standardised total and indirect effects between ecological factors under grazing conditions

Notes: pH: soil pH; MCS: microbial community structure, which is a dimensionality reduction value of F:B, MBC and MBN; AGB: aboveground biomass; BGB: belowground biomass; PD: plant diversity; SN: soil nutrients, which is a dimensionality reduction value of SOC, TN, TP, C:N, C:P and N:P.
